# Design and Psychometric Evaluation of Nurses’ Mobile Health Device Acceptance Scale (NMHDA-Scale): Application of the Expectation-Confirmation Theory

**DOI:** 10.2196/55324

**Published:** 2024-09-17

**Authors:** Narjes Mirabootalebi, Zahra Meidani, Hossein Akbari, Fatemeh Rangraz Jeddi, Zahra Tagharrobi, Walter Swoboda, Felix Holl

**Affiliations:** 1 Health Information Management Research Center (HIMRC) Kashan University of Medical Sciences Kashan Iran; 2 Department of Health Information Management & Technology Allied Medical Sciences Faculty Kashan University of Medical Sciences Kashan Iran; 3 Social Determinants of Health Research Center Kashan Iran; 4 Trauma Nursing Research Centre Kashan University of Medical Sciences Kashan Iran; 5 DigiHealth Institute Neu-Ulm University of Applied Sciences Neu-Ulm Germany

**Keywords:** mobile health, acceptance, psychometric evaluation, nursing, Expectation-Confirmation Theory, smartphone

## Abstract

**Background:**

The use of mobile tools in nursing care is indispensable. Given the importance of nurses’ acceptance of these tools in delivering effective care, this issue requires greater attention.

**Objective:**

This study aims to design the Mobile Health Tool Acceptance Scale for Nurses based on the Expectation-Confirmation Theory and to evaluate it psychometrically.

**Methods:**

Using a Waltz-based approach grounded in existing tools and the constructs of the Expectation-Confirmation Theory, the initial version of the scale was designed and evaluated for face and content validity. Construct validity was examined through exploratory factor analysis, concurrent validity, and known-group comparison. Reliability was assessed using measures of internal consistency and stability.

**Results:**

The initial version of the scale consisted of 33 items. During the qualitative and quantitative content validity stage, 1 item was added and 1 item was removed. Exploratory factor analysis, retaining 33 items, identified 5 factors that explained 70.53% of the variance. A significant positive correlation was found between the scores of the designed tool and nurses’ attitudes toward using mobile-based apps in nursing care (*r*=0.655, *P*<.001). The intraclass correlation coefficient, Cronbach α, and ω coefficient were 0.938, 0.953, and 0.907, respectively.

**Conclusions:**

The 33-item scale developed is a valid and reliable instrument for measuring nurses’ acceptance of mobile health tools.

## Introduction

Mobile phones and other electronic devices are becoming increasingly important for health care professionals [1]. These tools, along with mobile apps, provide numerous benefits for health care professions [2], including time savings [3]; cost-effectiveness [4]; enhanced self-efficacy [5]; greater access to evidence-based resources [6]; reduced medication errors [5]; decision support [6]; medication guides and guidelines [5]; video consultations with other physicians, alerts, and patient education [7]; and improved communication [5]. Given that the majority of hospital staff are nurses [8], and with an estimated 140,000 nurses in Iran according to a 2018 report by the Ministry of Health [9], it has been reported that approximately 80% of these nurses use mobile phones [8]. Another study found that 98% of nursing students use mobile phones to access drug guidelines, and 83% use them to look up medical terminology [10]. Unfortunately, hospitals do not provide personal mobile phones, and their use may raise concerns for health care organizations, potentially prompting them to implement policies to restrict mobile phone usage [11]. These concerns include distractions for nurses [12], infection control issues [13], and patient privacy risks [14]. Given the importance of mobile phones in nursing practice and the large population of nurses [15], health care stakeholders, managers, and planners should develop policies that support the appropriate use of mobile phones in nursing care and ensure its continuity [16]. The first step in achieving this is to assess the current situation, which requires a suitable tool. This tool should propose and validate a comprehensive model that considers individuals’ characteristics, technology, and tasks. The model should address factors such as user satisfaction, confirmation, mobile health (mHealth) continuance, maturity, mobility, individual performance, perceived usefulness, and personal habits [17]. In similar studies, tools specifically designed to identify the unique needs of different stakeholders—such as physicians, nurses, and patients, who have distinct priorities and requirements for using mobile tools—have not been developed. Many of these tools are general questionnaires used in health care and other industries, such as mobile banking and e-commerce, rather than being tailored specifically for nursing [18]. Despite the emphasis on the sociotechnical perspective, which highlights the importance of identifying behavioral and social factors alongside technical ones among users of information technology tools [19], the tools used in these studies often fail to adhere to appropriate psychometric principles. Moreover, they frequently overlook essential background, individual, social, organizational, and cultural factors in their design [20]. Research studies have yet to identify a tool with a suitable theoretical framework specifically designed and localized for nursing based on research and psychometric principles. Additionally, some health care organizations are actively working to establish mHealth stations. Therefore, this study aimed to design and psychometrically validate a Mobile Health Acceptance Tool for clinical nurses in Iran, based on the constructs proposed in the Expectation-Confirmation Theory. According to behavior change theories such as the Expectation-Confirmation Theory, nurses who have a positive perception of using mobile devices in health care, and who believe in their usefulness, effectiveness, and ease of use, are more likely to adopt mHealth tools. The concept of acceptance is explained through the constructs of the Expectation-Confirmation Theory. The scale measuring nurses’ acceptance of mHealth tools includes questions addressing various dimensions of attitude, belief, and intention to use mobile tools in providing nursing services, based on Expectation-Confirmation Theory structures. The study’s key strength was the application of the Expectation-Confirmation Theory to define acceptance of usage behavior, with a focus on the principles and stages of tool design according to the theory. Additionally, the study considered diverse research units based on individual characteristics, particularly employment context.

## Methods

### Overview

The researchers initially aimed to identify factors influencing nurses’ adoption of mobile devices using the Expectation-Confirmation Theory. They identified key factors such as security risk, new technology anxiety, subjective norms, perceived ease of use, and approval. Following this, they reviewed other studies on mobile app evaluation and developed a preliminary list of measurement items.

This research used a tool design and psychometric validation approach, which was conducted in 2 phases.

### Preliminary Phase: Designing the Initial Version of the Tool

In the preliminary phase, the tool was designed using the 4-stage approach proposed by Waltz et al [21]. First, the concept to be measured and its constructs were identified based on the Expectation-Confirmation Model. Second, measurement objectives were established based on the characteristics of the acceptance concept in the Expectation-Confirmation Theory, focusing on 8 constructs: perceived ease of use, social influence, new technology anxiety, personal habit, perceived security risk, confirmation, maturity, and perceived usefulness. In the third stage, the initial draft of the tool was developed. This involved a comprehensive search of both Persian and English articles in national and international databases, including PubMed, Ovid, Scopus, Web of Science, Magiran, IranDoc, Noor Mags, Science Direct, Jihad Daneshgahi Scientific Information Center, ProQuest, CINAHL, and SAGE, without time restrictions. The search utilized keywords related to mHealth, nursing, acceptance, scale, and attitude. All articles were critically reviewed to identify scales used to measure the acceptance of mHealth tools. Items from existing tools, including perceived usefulness, user satisfaction, and acceptance [22-25], were gathered from relevant texts, reviewed, categorized, and then integrated. Overlapping and inappropriate items were eliminated, and the research team determined the appropriate number of items for each decision domain. In the fourth phase, the tool’s development involved defining the wording of items based on the conceptual constructs of the theory. The researchers formulated item wordings according to the theory’s conceptual structures, revised them, and established scoring rules. Considering the cultural context of Iranian society and the organizational structure of Iranian hospitals, the research team reviewed and adjusted the items designed based on existing tools.

For item scoring, a 7-point Likert scale (ranging from 1=completely disagree to 7=completely agree) was used, in line with common principles in attitude measurement tool design. Reverse scoring was applied to negatively phrased questions [26]. The total score of the tool was calculated as the average score of all items.

### Phase 2: Psychometric Validation of the Tool

#### Step 1: Face and Content Validity Assessment

The preliminary questionnaire was reviewed by 10 experts in nursing, tool design, psychometric validation, health information management, and health informatics to assess qualitative content validity. Initially, a qualitative approach was used to gather expert opinions on the questionnaire’s comprehensibility, grammar, language, scoring, key aspects, essential components of the concept, and the clarity and simplicity of the items [27]. Based on their feedback, necessary revisions were made to the instrument.

For quantitative content validity assessment, the content validity ratio CVR_strict_, content validity index (CVI), and modified Kappa statistic (modified Kappa) were used. CVR_strict_ was calculated for each item, considering its necessity. Additionally, CVI and modified Kappa were calculated for each item based on the relevance criterion [28]. The Lawshe table, Waltz and Bausell index, and Polit and Beck criteria were used to evaluate the results for CVI, CVR, and modified Kappa. Additionally, the overall CVI for the entire instrument was calculated using the S-CVI_Average_ method [29].

To assess face validity qualitatively, in addition to the expert review, the first author (NM) read each question in the questionnaire aloud to 10 clinical nurses. Their interpretations of each question were compared with the original intent. The research team revised items in cases of ambiguity, inconsistency, or difficulty understanding the questions [30]. A professional Persian language editor was also consulted during this phase.

For the quantitative face validity assessment, nurses provided individual opinions on the importance of each item. They rated each item’s importance on a 5-point Likert scale. Based on these ratings, an item impact score was calculated for each item, with scores above 1.5 considered desirable [31].

#### Step 2: Questionnaire Item Analysis, Construct Validity (Factor Analysis, Concurrent Validity, and Comparison of Known Groups), and Ceiling and Floor Effects

Experts recommend a sample size of 100-300 individuals for psychometric validation of a tool, regardless of the number of items [31]. Therefore, this study targeted a sample size of 250 individuals. The inclusion criteria were working in clinical settings (direct patient care), a minimum of 6 months of clinical experience, holding a university degree in nursing, no known psychological disorders, Iranian citizenship, and consent to participate. The exclusion criteria were unwillingness to continue cooperation or withdrawal from completing the questionnaire during the study.

After assessing the face and content validity of the instrument and obtaining the necessary permissions, the first author (NM) visited the nursing offices of hospitals and conducted sampling with an introduction letter. The sampling was performed in a stratified random manner between 2021 and 2022, based on the type of clinical ward. Different clinical wards within hospitals under the coverage of Kashan University of Medical Sciences were identified, and a list of qualified nurses in those wards was compiled. A simple random sample of participants was selected from each ward using a random number table, in proportion to the required sample size and the number of nurses employed in each ward. A questionnaire was used to collect demographic data, including personal and occupational information such as age, gender, education, marital status, work experience, ward of employment, predominant shift, positions in nursing management, smartphone ownership, type of smartphone operating system, daily internet usage duration, and smartphone usage duration. This was accompanied by the initial version of the Mobile Health Acceptance Tool (validated in the final phase of face and content validity assessment) and a single-item tool to assess nurses’ attitudes toward using mobile-based apps in nursing care, rated on a scale from 1 (completely disagree) to 7 (completely agree). At the beginning of each shift, the ward was visited. After obtaining the nurses’ consent and providing general instructions on completing the tools, the questionnaires were collected at the end of the shift. If a questionnaire was not completed on time, arrangements were made with the respective nurse. If access was impossible or cooperation was not obtained from the selected individual, a replacement was randomly chosen from the same ward.

After collecting the data, item analysis was initially performed using the loop method. Exploratory factor analysis was then conducted using the maximum likelihood method with varimax rotation. Eigenvalues greater than 1 and scree plots were used to determine the number of factors. A factor loading above 0.44 was used as the threshold for item retention. Items were assigned to the factor that conceptually aligned with them based on their common factor loads. After conducting the factor analysis, ceiling and floor effects were evaluated. The instrument’s ceiling and floor effects were assessed based on the relative frequencies of samples with the highest and lowest achievable scores [32].

Concurrently, the known-groups comparison method was used to assess the construct validity of the final version of the instrument. Nurses were categorized into 7 groups, ranging from “1=completely agree” to “7=completely disagree,” based on their responses to a question evaluating their attitude toward using mobile-based applications in nursing care. The acceptance scores on the Mobile Health Tool Acceptance Scale were then compared across these groups.

#### Step 3: Reliability Assessment

The internal consistency of the final version of the instrument and its subscales (factors extracted during factor analysis) was assessed for the entire sample using Cronbach α coefficient and McDonald ω.

The test-retest method [33] was used to assess the instrument’s stability. Ten randomly selected participants from the study completed the final version of the instrument again 1 week later. The intraclass correlation coefficient between the scores from the 2 assessments was calculated, and standard error of measurement (SEM) and smallest detectable change (SDC) were also estimated [34].

### Data Analysis

Data analysis was conducted using SPSS version 16 (IBM Corp.). Quantitative variables were described using measures of central tendency and dispersion, while categorical variables were described using absolute and relative frequencies. Content validity was assessed quantitatively using CVI, CVR, and the modified Kappa statistic, and quantitative face validity was determined using the impact factor. The normality of quantitative data was assessed using skewness and kurtosis indices (for both parameters, the range of –2 to +2 was considered as the normal distribution). To check construct validity, exploratory factor analysis was performed using the maximum likelihood method with varimax rotation. The suitability of the data for exploratory factor analysis was evaluated using the Kaiser-Meyer-Olkin statistic and Bartlett test. The concurrent validity of the instrument was assessed using the Pearson correlation coefficient with the single-item attitude measurement scale. One-way ANOVA was used to compare known groups. The internal consistency of the instrument was assessed using Cronbach α and ω coefficients. Intraclass correlation coefficients were calculated to evaluate the correlation of scores between the 2 assessments in the test-retest. The SEM was computed using Equation 1, where SD represents the SD of scores and *r* is the Cronbach α coefficient.




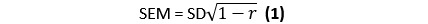




SDC was reported based on Equation 2. A significance level of <.05 was considered in all analyses.

SDC = 1.96 × √(2×SEM) **(2)**

### Ethics Approval

This study was approved by Kashan University of Medical Sciences (KAUMS), Kashan, Iran (ethical code number IR.KAUMS.NUHEPM.REC.1401.039).

## Results

### Preliminary Phase

The initial draft of the instrument consisted of 33 items across 8 domains: perceived ease of use, social influence, new technology anxiety, personal habit, perceived security risk, confirmation, maturity, and perceived usefulness. These domains included 5, 6, 4, 3, 3, 5, 3, and 4 items, respectively.

### Psychometric Phase

#### Stage 1: Content and Face Validity Assessment

In the qualitative content validity assessment, some items were revised. For example, the item “Nurses can easily use mobile app–based applications in patient care” was changed to “The interaction of nurses with mobile tools for providing nursing services is a simple task.” Additionally, the item “The use of mobile apps by nurses in patient care saves time” was added to the perceived ease of use domain.

In the quantitative content validity assessment, the CVR_strict_ for all items, except 1 that was removed, was equal to or higher than the acceptable value specified in the Lawshe table (the minimum acceptable CVR for 10 experts is 0.62). The CVI and the modified Kappa statistic for the 33 retained items were within the range of 0.80-1. Additionally, the S-CVI_Average_ was calculated to be 0.98.

In the face validity assessment, no changes were made to the items. Additionally, in the quantitative face validity assessment, the impact score for all items was above 1.5.

In summary, following the revisions in the initial psychometric phase, the final version of the instrument retained 33 items.

#### Stage 2: Questionnaire Item Analysis, Construct Validity (Factor Analysis, Concurrent Validity, and Comparison of Known Groups), and Ceiling and Floor Effects

During the sampling process, 357 (44.1%) eligible nurses out of 810 were selected. Of these, 107 (30%) did not consent to participate, resulting in data analysis for 250 (70%) individuals.

The mean age of the participants was 35.6 (SD 7.6) years, with an average work experience of 11.6 (6.7) years; 231 (92.4%) participants owned mobile phones, which they had used for an average of approximately 9.5 years (Table 1).

Item analysis revealed that removing items with correlation coefficients less than 0.30 or greater than 0.70 with the total score would not significantly impact the instrument’s α coefficient. Therefore, all items were retained.

**Figure 1:**
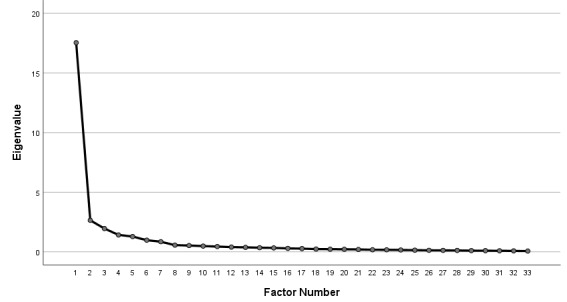
Scree plot of the Nurses’ Mobile Health Device Acceptance Scale (NMHDA-Scale).

The Kaiser-Meyer-Olkin measure was 0.943, and the Bartlett test of sphericity yielded a chi-square value of 8651.805 (*df*=528, *P*<.001), indicating the suitability of the 33-item instrument for factor analysis. All items had factor loadings above 0.44, and none were removed during this phase. Factor analysis extracted 5 factors that accounted for 70.539% of the total variance in the Mobile Health Tool Acceptance Scale score (see Tables 2 and 3, and Figure 1).

**Table 1:** Characteristics of clinical nurses working in hospitals under the coverage of Kashan University of Medical Sciences, 2022 (n=250).

Categorized variables		Values, n (%)
**Gender**		
	Male	44 (17.6)
	Female	206 (82.4)
**Marital status**		
	Married	201 (80.4)
	Single	47 (18.8)
	Divorced	2 (0.8)
	Widow	0 (0)
**Education**		
	Bachelor’s	217 (86.8)
	Master’s	33 (13.2)
	Doctorate	0 (0)
**Ward of employment**		
	Emergency	23 (9.2)
	Internal	31 (12.4)
	General surgery	50 (20.0)
	Intensive care unit	51 (20.4)
	Pediatrics	7 (2.8)
	Operating room	34 (13.6)
	Other	54 (21.6)
**Holding a position in nursing management levels**		
	Yes	49 (19.6)
	No	201 (80.4)
**Primary shift**		
	Morning	114 (45.6)
	Evening	29 (11.6)
	Night	36 (14.4)
	Rotating	71 (28.4)
**Having a smartphone**		
	Yes	231 (92.4)
	No	19 (7.6)
**Mobile operating system (if smartphone; n=231)**		
	Android	207 (89.6)
	Apple iOS	24 (10.4)
**Duration of internet usage during the day**		
	Less than 1 hour	49 (19.6)
	1-2 hours	91 (36.4)
	2-4 hours	62 (24.8)
	More than 4 hours	48 (19.2)

**Table 2:** Eigenvalue, explained variance percentage, and internal consistency coefficients of the factors extracted from the Mobile Health Tool Acceptance Scale in nurses along with their correlation coefficients with the single-item attitude assessment tool score.

Factor	Question number	Special value	Percentage of variance^a^	Internal consistency coefficient		Correlation with the single-item attitude assessment tool score		
				Cronbach α^b^	ω^c^	Pearson coefficient^d^	*P* value	
Factor 1	10	6.703	20.313	0.882	0.923	0.794	<.001	
Factor 2	7	4.671	14.155	0.916	0.919	0.642	<.001	
Factor 3	5	4.378	13.268	0.935	0.935	0.537	<.001	
Factor 4	7	3.844	11.469	0.950	0.950	0.591	<.001	
Factor 5	4	3.681	11.155	0.907	0.907	-0.447	<.001	

^a^The total percentage of variance explained by each factor was 70.539.

^b^The total value was 0.938.

^c^The total value was 0.953.

^d^The total value was 0.655.

**Table 3:** Items of the extracted factors in the factor analysis of the Mobile Health Tool Acceptance Scale and their factor loadings^a^.

Item number	Item	Extracted factor number^b^					
		1	2	3	4	5	
23	Using the mobile app–based tools in nursing care, beyond nurses’ expectations, helps in team coordination in processing patient information and making appropriate decisions.	0.818	—^c^	—	—	—	
22	Using the mobile app–based tools in nursing care, beyond nurses’ expectations, contributes to the improvement of care quality.	0.803	—	—	—	—	
24	Using the mobile app–based tools in nursing care, beyond nurses’ expectations, accelerates the execution of therapeutic and care interventions.	0.760	—	—	—	—	
26	Using the mobile app–based tools in nursing care, beyond nurses’ expectations, assists in the proper and effective implementation of clinical care guidelines and instructions.	0.741	—	—	—	—	
25	Using the mobile app–based tools in nursing care, beyond nurses’ expectations, enhances the management of nursing services.	0.739	—	—	—	—	
27^d^	Nurses can use the mobile app–based tools for their primary duties.	0.576	—	—	0491	—	
19	The more confident nurses are about the security of patients’ information when using the mobile app–based tools for nursing care, the more they use them.	0.573	—	—	—	—	
20	Nurses are willing to use the mobile app–based tools for care, provided they are confident that patient or hospital data are not accessible to unauthorized individuals.	0.564	—	—	—	—	
18	Nurses must use the mobile app–based tools for nursing care.	0.499	—	—	—	—	
21	The use of the mobile app–based tools in providing nursing care increases the potential risk of unauthorized individuals tampering with patient or hospital data.	0.452	—	—	—	—	
17	Nurses prefer to use the mobile app–based tools in patient care.	0.442	—	—	—	—	
8	Senior hospital and university managers support the use of the mobile app–based tools by nurses.	—	0.783	—	—	—	
9	Nurses recommend and emphasize the use of the mobile app–based tools for care to their colleagues.	—	0.736	—	—	—	
6	Nursing managers believe that nurses should use the mobile app–based tools in patient care.	—	0.690	—	—	—	
10	Physicians welcome the use of the mobile app–based tools in nursing care.	—	0.680	—	—	—	
11	Messages sent through social media and group media encourage nurses to use the mobile app–based tools in patient care.	—	0.595	—	—	—	
7	Higher authorities such as the Ministry of Health, Treatment, and Medical Education play a vital role in the use of the mobile app–based tools by nurses.	—	0.463	—	—	—	
16	Using the mobile app–based tools in patient care is considered normal among nurses.	—	0.442	—	—	—	
3	Learning how to use the mobile app–based tools for nursing care is easy.	—	—	0.797	—	—	
4	Gaining skills in using the mobile app–based tools for nursing care is easily possible.	—	—	0.770	—	—	
1	Nurses can easily use the mobile app–based tools for patient care.	—	—	0.755	—	—	
2	Nurses can perform patient care activities more easily using the mobile app–based tools.	—	—	0.739	—	—	
5	Nurses’ use of the mobile app–based tools in patient care helps save time.	—	—	0.686	—	—	
31	Using the mobile app–based tools in nursing care improves the process of collecting, documenting, and analyzing patients’ clinical data.	—	—	—	0.738	—	
33	Using the mobile app–based tools in nursing care enhances communication among nurses and other members of the health care team.	—	—	—	0.690	—	
32	Using the mobile app–based tools in nursing care supports family-centered care and reduces nurses’ direct involvement in some interventions, such as medication administration.	—	—	—	0.617	—	
30	Using the mobile app–based tools in nursing care increases nurses’ productivity.	—	—	—	0.612	—	
28^d^	The features of the mobile app–based tools are adaptable and compatible with nurses’ clinical performance.	0.525	—	—	0.543	—	
29^d^	The mobile app–based tools for assisting with daily nursing activities are sufficiently adequate.	0.488	—	—	0.527	—	
15	Nurses experience high stress when using the mobile app–based tools in patient care due to their inability to manage potential problems.	—	—	—	—	–0.864	
14	Using the mobile app–based tools in patient care confuses and bewilders nurses, making them feel disoriented.	—	—	—	—	–0.828	
12	Nurses have doubts about using the mobile app–based tools for patient care due to the fear of not being able to correct mistakes.	—	—	—	—	–0.719	
13	Mandatory use of the mobile app–based tools for patient care causes fear and anxiety in nurses.	—	—	—	—	–0.715	

^a^Factor naming is as follows: factor 1 encompasses 10 questions consisting of items 17, 18, 19, 20, 21, 22, 23, 24, 25, and 26, which were named “application and performance”; factor 2 encompasses 7 questions consisting of items 6, 7, 8, 9, 10, 11, and 16, which were named “social impact”; factor 3 encompasses 5 questions consisting of items 1, 2, 3, 4, and 5, which were named “perceived ease of use”; and factor 4 encompasses 7 questions consisting of items 27, 28, 29, 30, 31, 32, and 33, which were named “effectiveness”; and factor 5 encompasses 4 questions consisting of items 12, 13, 14, and 15, which were named “new technology anxiety.”

^b^A minimum factor loading of 0.44 was considered. Factor loadings less than 0.44 are not included in the table.

^c^Not available.

^d^For common factor loadings, the item was loaded on a factor that conceptually aligned with the item’s content.

Based on the instrument validated in the exploratory factor analysis (33 items), the mean Mobile Health Tool Acceptance Scale score for nurses was 4.207 (SD 0.740) on a 1-7 scale. With 95% CI, this score is estimated to range between 3.29 and 5.12 for the nursing population working in hospitals under the coverage of Kashan University of Medical Sciences. The mean score for nurses’ attitudes toward using the mobile app–based programs in nursing care, as measured by the single-item tool on a 1-7 scale, was 4.340 (SD 1.510). With 95% CI, this score is estimated to range between 2.468 and 6.212 for the target population. In the concurrent validity assessment, a significant positive correlation (*P*<.001) was found between the Mobile Health Tool Acceptance Scale score and the single-item tool score measuring nurses’ attitudes, indicating that higher acceptance scores were associated with more positive attitudes (Table 3). In the known-groups comparison, one-way ANOVA revealed a statistically significant difference in the Mobile Health Tool Acceptance Scale scores among groups based on their level of agreement or disagreement with using the mHealth tool (*P*<.001; Table 4).

In the floor and ceiling effect assessment, the relative frequencies of nurses’ lowest and highest possible scores on the Mobile Health Tool Acceptance Scale were both less than 15%.

**Table 4:** Acceptance Score of the Mobile Health Tool Acceptance Scale in nurses, differentiated by their overall agreement or disagreement regarding the use of the mobile app–based nursing care (single-item tool score; n=250)^a^.

One-way ANOVA results	Nurses’ agreement or disagreement with the use of the mobile app–based tools in nursing care (single-item tool)								Mobile Health Tool Acceptance Scale
	Completely disagree (n=13)	Disagree (n=22)	Somewhat disagree (n=27)	Neutral (n=64)	Somewhat agree (n=67)	Agree (n=43)	Completely agree (n=14)		
Score, mean (SD)	3.263 (0.782)	3.484 (0.833)	3.678 (0.546)	4.019 (0.324)	4.489 (0.443)	4.820 (0.436)	4.869 (1.195)	Welch statistic=31.266 (*P*<.001)	

^a^In terms of comparing the 2 groups, the Games-Howell post hoc test showed that there is a significant difference between the acceptance scores of the mobile health tool in the following group pairs: 1 and 7 (*P*<.006), 1 and 6 (*P*<.001), 1 and 5 (*P*<.001), 2 and 5 (*P*<.001), 2 and 6 (*P*<.001), 2 and 7 (*P*<.02), 3 and 5 (*P*<.001), 3 and 6 (*P*<.001), 3 and 7 (*P*<.03), 4 and 3 (*P*<.001), 4 and 5 (*P*<.001), and 6 and 5 (*P*<.004).

#### Stage 3: Internal Consistency and Stability

In the assessment of internal consistency, the Cronbach α coefficient and the ω total coefficient for the entire instrument were 0.938 and 0.953, respectively. The coefficients for the 5 extracted factors were also above 0.88 (Table 2).

In the tool’s stability assessment, the intraclass correlation coefficient between test and retest scores was 0.907 (95% CI 0.615-0.977, *P*<.001).

The SEM for the designed instrument was 0.184, and the SDC was 1.19, with 95% CI.

## Discussion

### Principal Findings

Previous studies have used measurement tools to assess the use of mHealth tools in various health care and other groups [18,35,36]. However, none of these studies utilized a psychometrically tested tool specifically designed for nurses. Therefore, this study aimed to develop and validate the Nurses’ Mobile Health Device Acceptance Scale (NMHDA-Scale) based on the Expectation-Confirmation Model.

A 33-item questionnaire was designed to assess the acceptance of mHealth tools among clinical nurses, demonstrating strong validity and reliability within the target population.

The draft of the NMHDA scale was developed using Waltz’s 4-step approach and the Expectation-Confirmation Model. This study, grounded in its theoretical framework and the principles of the Expectation-Confirmation Model [37], offers a broader range of acceptance dimensions compared with other existing tools [38,39]. Experts argue that a theoretical foundation for defining the content domains of a tool leads to the creation of relevant items and is crucial for ensuring the tool’s validity [40].

In the content validity assessment, revisions to the tool were made based on expert feedback from various relevant and specialized fields. The CVR, CVI, and modified Kappa statistics for all retained items were higher than 0.62, 0.80, and 0.74, respectively. Additionally, the overall S-CVI (S-CVI_Average_) for the entire tool was greater than 0.90. Experts agree that assessing content validity is crucial to ensure the tool covers all essential aspects of the intended concept. The reliability of this process increases with the expertise of the individuals involved in this stage [21]. Moreover, an S-CVI_Average_ greater than 0.90 is considered desirable for content validity [41]. Based on the presented information, the developed tool meets the criteria for establishing content validity.

In the face validity assessment, modifications were made to the tool based on feedback from clinical nurses, the target group. Additionally, the impact scores for all retained items were above 1.5. Connell et al [42] highlighted that face validity is crucial for addressing the needs of the target group, as what researchers consider essential may differ from the perspective of the primary group. Therefore, face validity can enhance the measurement’s acceptability, relevance, and quality [42]. Thus, it can be claimed that the tool’s items are well understood by the target group, confirming its face validity.

The exploratory factor analysis identified 5 factors: “application and performance,” “social impact,” “perceived ease of use,” “effectiveness,” and “new technology anxiety.” These factors collectively explained more than 50% of the total score variance, with each contributing over 5%. Additionally, all items had factor loadings exceeding 0.44, and there was one common factor among them. Some experts argue that for construct validity, the identified factors should account for at least 40% of the total variance [43], with each factor explaining more than 5% of the total variability [44]. Additionally, other sources suggest that for robust construct validity, the factors should collectively account for more than 50% of the total variance [45]. Given that the identified factors in this study explained over 50% of the total variance, each contributing more than 5%, the construct validity of the tool is well-established. Therefore, the exploratory factor analysis results suggest the construct validity of the tool. The high factor loadings of the items and the existence of only one common factor further support the desirable structure of the tool [46].

The content of the items loaded onto the extracted factors aligns well with the intended acceptance concept. For instance, factors such as “technology anxiety,” “social impact,” and “perceived ease of use” directly correspond with theoretical expectations. The factor “usefulness and performance” aligns with the “security risk” factor, while “confirmation” and “effectiveness” correspond with “maturity” and “perceived benefit.” Compared with other tools used to assess the acceptance of mHealth tools [47], the structure of this tool is notably more desirable.

A comparison of known groups revealed a significant difference in acceptance scores among nurses based on their agreement or disagreement with using mHealth tools. This finding indicates that the designed tool can effectively differentiate between nurses with varying levels of agreement regarding mHealth tools. Such a result supports the structural validity of the tool, as it is intended to distinguish between groups expected to have differences in a specific characteristic. The observed significant differences further validate the tool’s structure [48].

In our study, the relative frequency of the minimum and maximum possible scores obtainable from the acceptance measurement tool was 0, indicating the absence of floor and ceiling effects. Floor and ceiling effects are observed when more than 15% of respondents achieve the highest or lowest possible score on a tool. The absence of such effects in this study suggests that the tool’s items are appropriately distributed across the scale, supporting both the content validity and reliability of the instrument [49].

In our study, the total Cronbach α coefficient of the tool was 0.938. According to Shrestha [50], an acceptable lower limit for Cronbach α for reliability is 0.70. Values between 0.60 and 0.80 are considered average, while those between 0.80 and 1.00 are deemed very good [51]. Thus, the high Cronbach α value indicates that the tool demonstrates excellent internal consistency.

The correlation coefficient between the scores obtained from the 2 test sessions was 0.655. Lotfi et al [52] suggested that a correlation coefficient between scores from 2 test sessions indicates the test’s stability and repeatability, with coefficients above 0.70 considered acceptable and those above 0.80 very good. Although the coefficient in this study is slightly below the optimal threshold, it still reflects the tool’s satisfactory stability and demonstrates higher consistency compared with similar tools [39].

In our study, the SEM was estimated to be 0.184, with the SDC being 1.19. This means that if the test is repeated for an individual, their score may vary by up to 0.184 points. The small SEM supports the tool’s stability and reliability [53]. Given the tool’s score range, this SEM value indicates the tool’s robustness in terms of stability, repeatability, and overall reliability.

### Study Limitations

This study has several limitations: data collection during the COVID-19 crisis, which coincided with a high workload for nursing staff, led to a significant number of nurses being unwilling to cooperate. Additionally, the lack of appropriate Persian language tools for assessing convergent validity presents another constraint.

### Application of Findings

The current research serves as a valuable tool across various organizational dimensions. It assists senior managers and decision makers in evaluating the potential success of new technologies. For instance, it can contribute to reshaping nursing education curricula by integrating organizational and environmental variables, developing guidelines and regulations for app design, and more. This tool assists nurses in understanding the benefits of technology use by focusing on perceived usefulness, ease of use, adoption concerns, security perceptions, user satisfaction, habit maturity, acceptance, and societal impact. The integration of mobile tools among nurses underscores the need for national policies that provide a clear framework for the design, implementation, and evaluation of mobile apps in nursing.

### Conclusions

This study details the development and psychometric evaluation of the Mobile Health Tool Acceptance Scale for nurses, grounded in the Expectation-Confirmation Theory. Exploratory factor analysis confirmed a 5-factor model with 33 items. The factors—usability and performance, social influence, perceived ease of use, effectiveness, and anxiety about new technology—are distinct and collectively capture nurses’ intention to accept mHealth tools for professional and job-related purposes. Future studies can utilize this tool to assess the intention of nurses and other health care professionals to use mobile phones for work purposes. This approach allows for the exploration of predictors and outcomes related to both theoretical frameworks and practical applications.

This tool provides valuable insights for managers at various levels, aiding in the creation of guidelines and strategies related to the design and use of mobile apps in the health sector. It also supports the Ministry of Health and Medical Sciences Universities in revising nursing education curricula to enhance the integration of information technology tools and expand their usage based on key efficiency factors. Given that health service providers share common missions and goals and collaborate as a team during clinical activities, it is likely that, with appropriate modifications and psychometric testing, the revised scale could be adapted for use with other target groups within the health care field.

## Abbreviations

CVI: content validity index
CVR: content validity ratio
mHealth: mobile health
NMHDA-Scale: Nurses’ Mobile Health Device Acceptance Scale
SDC: smallest detectable change
SEM: standard error of measurement

## Data Availability

The developed Mobile Health Tool Acceptance Scale for Nurses is available from the corresponding author on reasonable request.
